# Innovative perception analysis of HIV prevention messaging for black women in college: a proof of concept study

**DOI:** 10.1186/s12889-022-13564-4

**Published:** 2022-06-25

**Authors:** Rasheeta Chandler, Henry Ross, Dominique Guillaume

**Affiliations:** 1 Nell Hodgson Woodruff School of Nursing, Emory University, Atlanta, GA USA; 2 Center for Community Practice, University of Rochester, Rochester, NY USA; 3 School of Nursing, Johns Hopkins University, Baltimore, MD USA

**Keywords:** HIV prevention, Black women, Health communication, Media messaging

## Abstract

**Background:**

Black women in college are disproportionately affected by HIV, but have not been a population of focus for HIV prevention campaigns. This study used content from a preexisting HIV media campaign to assess its relevancy and acceptability among Black women in college.

**Methods:**

Media viewing and listening sessions were convened with Black women enrolled at an HBCU (*n* = 10) using perception analyzer technology—hardware and software tools that are calibrated to gather and interpret continuous, in-the-moment feedback. Matched pre-and-post-test responses from focus groups were obtained from the perception analyzer data. Descriptive statistics and t-tests were used to characterize the data.

**Results:**

Students were more likely to personally identify with media content that included profound statements, along with memorable people and actors [95% CI: 1.38, 2.27]. In over half of the vignettes, participants reported that content representing students’ society, culture, or interests was missing.

**Conclusions:**

HIV prevention media campaigns may offer potential in increasing HIV awareness and risk perceptions; further research is needed to evaluate optimal content tailoring for both cultural and climate relevancy.

**Supplementary Information:**

The online version contains supplementary material available at 10.1186/s12889-022-13564-4.

## Introduction

Black women in college are disproportionately overrepresented by HIV incidence rates compared to their White counterparts [1, 2]. While studies have demonstrated that Black women in college have higher HIV knowledge rates compared to White students [3, 4], many Black women in college have low awareness and perceptions of their HIV risk [4–8]. As a result, despite having higher HIV knowledge rates, Black women in college who may not perceive themselves as being at risk may engage in behaviors that may increase their susceptibility to HIV infection. This suggests that solely imparting HIV knowledge may be insufficient and ineffective in reducing HIV rates among this group. Interventions that not only impart knowledge, but also increase HIV risk perception and awareness of situations that pose significant risk [3, 4] are desparately needed. There has been a scarcity of research assessing HIV prevention interventions for young Black women in college that specifically focus on HIV risk perception and awareness using mass media campaigns. Given this dearth in research, there is a need for the development and implementation of interventions that highlight Black college women’s HIV risk through targeted and relatable messaging.

Mass media interventions have the potential to reach large audiences and can be tailored to provide evidence-based sexual health and HIV risk reduction content that is culturally and contextually appropriate for Black women [9]. Media interventions that have targeted adolescents show greater sexual risk reduction within the greater community, and shifts in individual HIV/AIDS perceptions and attitudes [10, 11]. HIV prevention mass media campaigns directly targeting Black women in the U.S. have been remarkably limited. Today, there still are no HIV mass media campaigns designed specifically for Black women in college. Increased HIV knowledge coupled with media messages that transform perceptions and attitudes, can result in the reduction of sexual HIV transmission and health promotion behaviors such as frequent testing [10, 11]; an engagement opportunity that is widely thought of as a gateway to health services for many with limited health access.

### Project iMPPACS and other media messaging

Project iMPPACS [12] implemented by Sznitman and colleagues [9] was a multi-site mass media campaign using television and radio advertisements containing culturally and developmentally sensitive HIV prevention information for Black adolescents of low socioeconomic status (SES). The mass media campaign aimed to increase HIV transmission perception among Black adolescents with outcomes that included increasing condom usage and reducing HIV incidence-related behaviors [9, 13]. Project iMPPACS demonstrated influence in risk reduction behavior change, and change patterns, among the priority population [9, 13]. However, it is unclear if the messaging and images used in this intervention would resonate specifically with Black women in college.

HIV prevention initiatives typically target communities of low socioeconomic status and, while this work is necessary given their risk, Black women in college and of higher socioeconomic statuses are often not considered in the development of such interventions despite experiencing similar high HIV risks. Given the lack of attention placed on this population, Black women in college may not relate to media messages targeting lower income Black populations due to differences in lived experiences [14]. Partnering with Black women who attend college to conceptualize and design Sexual/HIV risk reduction communication might render the greatest degree of relatability among Black college women. The purpose of this study was to examine appropriateness, acceptability and relevance of Project iMPPACS advertisements and other publicly available audio-only media (Public Service Announcements [PSAs] among Black college women. Our aim was to have participants identify specific prevention-related content within the HIV prevention laden advertisements they found to be appealing, unpleasant, or critically important in efforts to inform and develop tailored messaging. Perception analyzers were used to record in real-time participant attitudes and reactions to media messages, and the feasibility of using this novel technology was explored. Explicit identification of relevant and health-motivating content may inform future message design, production, and dissemination for this population.

### Theoretical framework

This study was guided by the Social Norms Theory developed by Perkins and Berkowitz (1986) [15]. Social norms have remained a fundamental part of creative communications strategies particularly in health and entertainment education. This theory is widely used in promoting positive health-related behaviors and operates under the premise that individuals misperceive their peers’ behaviors and attitudes, and under-and-over estimate behaviors and peer approval [16]. This theory consists of six constructs: 1) descriptive norms (beliefs about what other people do) 2)injunctive norms (beliefs about what others approve of/think people should do) 3)reference group (a group of people that a person feels a connection or identity with) 4)sanctions and punishments (beliefs about the perceived sanctions/punishments) 5)benefits and rewards (beliefs about the perceived benefits/rewards) 6) collective norms (actual prevalence of a behavior) [17]. In addition, certain advertisements involved Black men and women in the community discussing the importance of safe sex and reducing HIV risk. Undertaking these approaches were largely aimed to normalize safe sex, address misperceptions regarding sex in the community, and promote condom use to reduce HIV risk within the Black community. Social norms theory has been used to guide social marketing campaigns targeted at the group level, with the use of mass media to disseminate social normative feedback [16]. However, literature on social norms in increasing health promotion behaviors has been relatively mixed,with certain studies demonstrating significance in behavior change and misperceived norms and other studies not demonstrating any significance [16]. While this theory has been widely used in health behavior interventions, literature has been limited on how social norms theory can be used to guide health promotion interventions that use digital technology and digital media. Furthermore, there is a need to understand how this theory can be used and adapted to guide studies within communities such as Historically Black Colleges and Universities (HBCUs) in which the majority of the population are young adults who identify as Black. This study focused on the constructs of descriptive norms, injunctive norms, and reference groups. The Project iMPACCS vignettes and PSAs were developed for Black youth to encourage HIV and STI (sexually transmitted infection) risk reduction through the use of strategies like message delivery by influential members of the Black community (e.g. popular artists and actors within the Black community). It was unclear whether the content in Project iMPACCS or the selected publicly available PSAs would be effective among Black women in college.

## Methods

### Participant criteria

Black women who were enrolled as either full or part-time students at a southern HBCU were invited to participate. All participants were required to self-report their race as Black, have no auditory or visual impairment, and speak and understand English. Additionally, participants had to be 18–24 years old, previously or currently sexually active within 6 months, and had to have reported media consumption (e.g., radio, television, social media, other audio/visual media) for at least one hour per day.

### Recruitment

A convenience sample was obtained through face-to-face, campus (e.g., dining halls), and electronic newsletter distributions of a study flyer. This research opportunity was also presented to various academic colleges (e.g., Media & Technology) and student organizations within the university. The creatively-designed advertisement included the pre-determined focus group schedule, participant incentive, and study contact information. A pre-screening script was used to guide determine study eligibility. If all criteria were met, participants were given the opportunity to reserve attendance for an open session. This schedule was updated daily during active enrollment. Participants who contacted study staff by phone or email received a return reply within one business day. The pre-screening script was also used to facilitate enrollment by phone. Students who expressed interest after the focus group was closed to enrollment were asked to provide their name and contact information in the event of cancellations. This information was destroyed upon study completion. Each participant received weekly telephone and email reminders of their study appointment (i.e., location and date/time). Plans for attendance were confirmed one day prior to their focus group session. Participants were also notified that, upon arrival, they would be required to sign a consent form which explains the study and its potential risks/benefits, confidentiality, and voluntary withdrawal. A copy of this consent was emailed to each participant for advanced review.

### Instruments

#### Perception analyzers

Perception Analyzer devices were used to provide participants with the ability to individually answer questions, while offering privacy and anonymity. This tool allows for participants to answer questions more honestly with continuous in-the-moment feedback and has been demonstrated to increase cognitive engagement within research interventions [18]. Given these advantages, perception analyzers hold the ability of gathering accurate data and reducing the impact of group dynamics such as reducing group think and social desirability bias [19]. This tool, while being primarily used within political science research, to our knowledge has not been used within public health research. In designing media campaigns for Black women, this tool holds significant potential in providing an objective method for identifying and assessing key messages that may be effective in eliciting emotional responses. Perception analyzers developed by Dialsmith™ were used to measure the responses of our study participants as they viewed and the Project iMPPACS vignettes and listened to the PSA radio messages.

#### Additional study questions

Additional study questions were developed by the research team through using preliminary research assessing the impact of HIV prevention content on sexual risk behavior [20–22] along with preliminary research evaluating Black women's perceptions of HIV prevention content and the influence of media on their sexual health needs [9, 23, 24].

### Study Outcomes

The primary outcome measure of this study was to evaluate the acceptability of the Project iMPPACS and publicly available audio-only HIV prevention messages among Black college women, and whether students believed the content would be beneficial in promoting safe sexual behaviors. The secondary outcome was assessing the feasibility of using perception analyzers in recording participant attitudes and reactions to Project iMPPACS and other media messages in real-time.

### Study procedures

The study was conducted in a lecture hall on a southern HBCU campus. Participants were introduced to the study staff and the Dialsmith administrator, followed by a brief overview of the study. The informed consent document was then reviewed; participants were given the opportunity to ask questions, summarize key principals in their own words, or exit the perception analysis session if they chose not to participate. All remaining participants then signed the informed consent document; these were collected and verified prior to the start of the focus group discussion. The Dialsmith administrator then distributed a perception analyzer tool to each participant. Each tool was labeled with a unique number that allowed study staff to identify each individual (along with self-reported classification and sexual activity) as well as their synchronous feedback. Prior to showing the Project iMPPACS media content or playing the PSAs, participants were asked questions pertaining to demographic data along with media intake. Participants were also asked a set of warm-up questions to test the perception analyzer for accuracy.

#### Perception analyzer implementation

We employed a DialSmith representative to explain and assist in use of perception analyzer devices and software for each session. The study space was arranged in a U-shaped configuration with 12 participant stations (each with a numerically coded perception analyzer device), and a central table reserved for synchronous monitoring and computing by the study team and DialSmith. The participant stations were spaced equally apart to provide privacy (the perception analyzer viewing screen is only approximately 2” wide) and clearance for the input cords.

In each session, the DialSmith representative introduced the perception analyzer to participants and discussed how it would be used. Participants were asked to place the perception analyzer tool in their non-dominant hand, and note two items – a viewing screen and a small mechanical dial. The dial was set to midpoint 50 (minimum dial value 0, maximum dial value 100). We explained the 50 would be used as a starting point or baseline, and that participants could show their approval by turning the dial to the right (“dial up”), or disapproval by turning the dial to the left (“dial down”) using their dominant hand. A number of non-study related iMPPACS advertisements were used as practice for participants to familiarize themselves with the smoothness and speed at which the dial responded to adjustments.

#### Media content

Media content was divided into two groups- television vignettes and radio advertisements- with a total of 4 TV vignettes and 2 radio messages (Table 1). Prior to playing the individual vignettes and radio media content, participants were asked pre-test questions (e.g., What content in today’s visual (e.g., TV, internet, videos) and audio (e.g., radio) commercials do you remember most?). Post-test questions ascertained Black college women’s views of the Project iMPPACS and PSA content (e.g., What content in this video or audio message do you remember the most? How do you personally identify with the content in this message? What is missing from the message that could influence you to engage in safer sex? How helpful would this message be if broadcasted to your preferred viewing stations?). Questions were asked as to whether participants perceived the content as being relevant for Black college women students, and whether additional sexual health content should be included. Participants were also asked to indicate their preferred method of content delivery [e.g., radio (audio only), TV (audio-visual), internet (portable: audio-visual)]. See the electronic supplementary materials for a full list of pre-and post-test questions.

**Table 1:** Descriptions of iMPPACS audiovisual and audio-only vignettes reviewed in focus group sessions [23]

Media title	Context/setting	Characters/interactions	HIV prevention and risk perception/ reduction content
*Audiovisual Vignettes*			
“Spot 3— Relationship”	Outdoors (street, park)	Individual interviews: two young adult Black males, two young adult Black females	Sexual communication (partner), sexual pressure (partner), abstinence
“He’s Experienced”	Transitional settings: from high school to health clinic	Dialogue between two young adult Black females (friends)	Condom negotiation, sexual communication (partner, peer)
“Class of 2008”	Graduation ceremony: announcement of graduates	Spotlighting of several individual graduates; one young adult Black male referenced for popularity and STI infection	STI infection, influence of social popularity on sexual behavior
“Check Yourself”	Night club	Dialogue between young adult Black male/ female	HIV disclosure, HIV stigma, condom negotiation, sexual communication (partner)
*Audio-only Vignettes*			
“Best Friends”	Telephone conversation	Dialogue between male and female	Condom negotiation, sexual communication (partner, peer), risk assessment
“I’ve Got Mine”	House party	Sexual encounter between male and female	Condom negotiation, sexual communication (partner), sexual trust, self-efficacy/respect
“Girls Who Respect Themselves”	Private (one on one)	Male seeks advice from another popular (referenced) male	Self-efficacy/respect, sexual communication (partner, peer), condom negotiation

### Data Analysis 

The perception analyzer responses from the focus groups were coded and exported as a Microsoft Excel spreadsheet. The baseline demographics of the sample were characterized using measures of central tendency. Matched pre-and-post-test responses were obtained from the perception analyzers and used for data analysis. Descriptive statistics and t-tests were used to characterize the data, with Type 1 error rate for statistical tests being set at 0.05 for significance and 95% confidence intervals provided where appropriate.

## Results

### Demographics

A total of 10 students participated in the study. The mean age of study participants was 19.5 with ages ranging from 18–22 years old. Participants largely held Freshman classification (*n* = 6, 60%) with the majority of students reporting that they were currently sexually active (*n* = 6, 60%). Among those who were sexually active, 90% (*n* = 9) reported being sexually active with men see Table 2.

**Table 2:** Participant demographics

Characteristics	*n* = 10 (%)
**Age**	19.5 (mean)
Classification	
Freshman	6 (60%)
Sophomore	1 (10%)
Junior	2 (20%)
Senior	1 (10%)
**Currently Sexually Active**	
Yes	6 (60%)
No	4 (40%)
**Sex of partners**	
Men	9 (90%)
Women	0 (0%)
Both	1 (10%)
**Sexually Active with male partner in past 3 months**	
Yes	6 (60%)
No	4 (40%)

### Media intake

Participants reported watching or listening to social media more often over radio and print media (77.8±17.01). When asked about whether participants watched or listened to television or radio more frequently, participants reported watching television more frequently over listening to the radio (23.1±16.51; 95% CI 11.29,34.91). Participants were more likely to state that HIV topics pertaining to risk, exposure, testing, and disclosure were more likely to be discussed in their current sexual situations as opposed to STI topics; and that condoms and other family planning options presented in media would have the most influence on their immediate decision to have protected sex (78.9±17.96; 95% CI 66.05,91.75) (Table 3).

**Table 3:** Media intake questions

	**M (SD)**	**95% CI**
Media Intake		
Which of these forms of media do you watch and/or listen to most often? Radio OR Social Media?	77.8 (17.0)	65.63, 89.97
Which of these forms of media do you watch and/or listen to most often? Social Media OR Print Media?	20.9 (18.69)	7.53, 34.27
Which of these forms of media do you watch and/or listen to most often? Television OR Radio?	23.1 (16.51)	11.29, 34.09
How many hours do you watch TV or other audio-visual media formats (like YouTube) per day?	6.8 (2.62)	4.93, 8.67
How many hours do you listen to the radio or other audio-only media formats per day?	8.3 (6.45)	3.69, 12.91

#### Integrated synchronous data presentation made available in digital form

The following YouTube playlist link—CRAZE Data Presentation (https://www.youtube.com/channel/UCnrp6T9yQLB37GYrArjjCoQ/videos)—contains a selection of video clips that were produced from the integrated/overlaid synchronous data capture of media perception, content and participant demographics for the duration of each advertisement. Clips are available from PSA audio message #1, and iMPPACS video messages #1–4. We present this resource as a visual immersive aid for readers to “see, hear and feel” as the participants did while the select advertisements were being played. A brief description (i.e., setting, music, actors) and examples of “dial up” / “dial down” intervals are provided (Additional file 1). Viewers are encouraged to use these data presentations in recognizing potential influencing factors related to this concept.

### HIV video vignettes

The degree to which participants identified with the vignettes’ content ranged from slightly to moderately well [1.25±1.39; 95% CI 0.09, 2.41] for the first vignette, and moderately well for both the second [2±1.70; 95% CI 0.78,3.22] and the third vignette [2.1±1.53; 95% CI 1.01, 3.19]. Participants reported greater and more intense personal connections with the fourth vignette compared to the other vignettes [3.88±0.36; 95% CI 3.58, 4.17]. Participants reported that the content they remembered the most in the fourth vignette were main discussion points (*n* = 6, 75%). Additional content that participants remembered the most within the other vignettes included profound statements (*n* = 5; 50%) followed by people and actors (*n* = 3; 30%) and main discussion points (*n* = 3, 30). Participants had higher average responses for the fourth vignette regarding their immediate interest in learning more about the topics discussed, with participants wanting to learn more about the fourth vignette compared to the other vignettes [2.34±1.38; 95% CI 1.38, 2.27].

Among the vignettes, participants were more likely to recommend the fourth vignette to their close friends and family [3.5±0,76; 95% CI 2.87,4.13] and believed that the video would be somewhat effective to very effective if shown on campus [2.62±1.19; 95% CI, 1.632, 3.618] and if broadcasted on their preferred viewing stations [3.63±0.74; 95% CI 3.00, 4.25]. For each of the vignettes, the large majority of participants responded that if they were to watch a similar video advertisement before watching sexually-charged visual media, then it would make them more aware of their sexual practices and any potential risks (*n* = 3, 30% for 1^st^ video message; *n* = 6, 60% for 2^nd^ video message; *n* = 6, 75% for 3^rd^ video message; *n* = 5, 62.5% for 4^th^ video message). Students were asked what content was missing from the vignettes that could influence them to engage in safer sex. In over half of the vignettes (75%) participants reported that content representing students’ current society, culture, or interests was missing. Additional content that students reported to be missing included “catch” phrases (e.g., hook lines and punch lines), profound statements, and songs, jingles, and music.

### HIV radio messages

Responses largely varied in regard to how participants personally identified with audio message content, with average responses ranging from slightly well to very well across the individual audio messages. Participants were more likely to recommend the audio messages that they strongly identified to their friends and family (2.5±1.69; 95% CI 1.09,3.91) in comparison to audio messages that they did not identify with strongly. Additionally, participants reported that the messages they personally identified with more would be between somewhat effective to very effective if they were to be broadcasted on campus and on their preferred listening stations. The majority of participants (*n* = 5, 62.5% for 1^st^ audio message; *n* = 6, 75% for 2^nd^ audio message) reported that the people and actors in the audio messages were the most memorable aspect of the advertisement, followed by profound statements (*n* = 3, 37.5%) and main discussion points (*n* = 3, 37.5%). Participants were less likely to be interested in learning more about the topics discussed in the audio message that they did not personally identify with strongly (1.5±0.76, 95% CI 0.87,2.13).

When asked about content that was missing from the audio messages that could influence participants to engage in safer sex, among all three of the audio messages participants reported content that represented their current society, culture, or interests was missing. Additional content that was reported to be missing were catch phrases and profound statements. In two of the audio messages, at least half of participants reported that if they were to listen to the audio message content before listening to sexually charged radio/songs, they would be more likely to participate in safer-sex activities (*n* = 5, 62.5% for 1^st^ audio message; *n* = 4, 50% for 2^nd^ audio message). Interestingly, in the third audio message which happened to be the message participants were least likely to identify with, half of participants responded that they would feel annoyed if they were to listen to the audio message prior to listening to sexually-charged radio/songs with only two (25%) of participants stating that the audio message would make them more aware of their sexual practices and risks.

### Feasibility of perception analyzers

Perception Analyzers were feasible to use in this setting and allowed for students to establish an objective and independent assessment of each HIV prevention advertisement, which was followed by more descriptive discussions of real-time outcomes from the perception analyzer data. Students appreciated the ability to give distinct feedback about varies content within the advertisement. For instance, the participant may like specific content or visuals in one portion of the advertisement and not in others. Using this method, we were able to get nuanced, objective data about each element of the advertisement (Additional file 1: Fig. 1).

## Discussion

Our study assessed the acceptability of Project iMPPACS’s and PSA HIV prevention messages for Black women through the use of innovative perception analyzer technology. Among the television vignettes, participants resonated stronger with the vignette that highlighted key discussion points. For the audio messages, participants were more likely to personally identify with content that included memorable people and actors within the advertisement. Among both the television vignettes and the audio messages, participants were more likely to recommend the content they strongly identified with to their peers. This finding aligns with prior research, which has demonstrated that mass media can elicit reciprocal communication. The emphasis on utilizing mass media in HIV campaigns to promote interpersonal communication among peers can play a significant role in promoting behavior change and increasing safe sex behaviors [25, 26]. Mass media campaigns that are effective in their content delivery and increasing awareness towards HIV can facilitate interpersonal communication and increasing engagement in discussions about HIV with peers [25–27]. Therefore, in facilitating interpersonal communication, it is imperative for individuals to personally identify with and relate to the content being discussed or displayed within advertisements. In our study, several participants reported resonating with profound message content within media advertisements. In health communication and promotion of uptake of prevention behaviors, message construction is critical in communication campaigns, with research highlighting key elements such as invoking emotional responses, narrative persuasion, visual representation of risk, familiarity, novelty, vivid content [25, 28]. Among the radio messages in particular, the use of important people and actors had a positive effect on participants relating or identifying more with the content. Advertising and marketing research have repeatedly shown that racial similarities between minority actors and minority viewers may result in greater message recall and favorable attitudes towards the advertised content [29]. Disease prevention messages using mass media that engages target audiences through memorable plots, appealing characters, with storylines can demonstrate effectiveness and potentially result in uptake of health promotion practices (i.e., condom use) [30].

The cultural complexities of media messages about HIV are rarely interrogated especially in the context of Black college women. The lack of content relatable to participants’ current society, culture, or interests appeared to be a reemerging theme among the media messages. Incorporating cultural factors are essential to HIV prevention efforts given that one’s culture can shape the way they view sex and engage in sexual behaviors; thus, identifying culturally sensitive messages that can potentially support safer sex behavior for this specific audience is critical [22]. The experiences of Black women in college are often not incorporated into HIV prevention messaging, with most mass media messaging campaigns targeting Black youth of low socioeconomic status. Given that data has repeatedly demonstrated that Black women encounter higher HIV transmission situations, there is a need for more diverse interventions that target Black women of differing backgrounds including those of varying socioeconomic statuses and education levels. Culture is not monolithic, nor does it exist in a vacuum; it is dynamic and exists on a continuum representing a wide range of beliefs and lived experiences and dominant norms. Although the original Project iMPPACS campaign was culturally tailored to address HIV prevention in Black youth, many of the women in our study felt that the content did not speak to their culture or lived experiences. Thus, this reiterates the notion that culture is complex and that developing media content for this population requires garnering an understanding of what speaks to their lived experiences. It is important to consider that our sample consisted of Black college students at an HBCU, and findings may have differed if we sampled Black students from a predominantly White University (PWI). Previous findings by the research team noted that while HIV-related behavioral patterns among HBCU and PWI Black female students are largely similar, certain socio-cultural factors tend to have higher predominance within HBCU campuses compared to PWI campuses[14]. Therefore, this may influence the effectiveness of health promotion messages in relation to content framing and delivery platforms. For instance, Payton et al. (2016) indicated that among Black female college students at two large universities were concerned about societal perceptions of being socially connected to HIV discourse even when the disease did not directly affect them [31]. Such findings need to be considered in the development of mass communication strategies particularly as more young adults use social media and internet-based platforms for health information [31, 32].

Most of the video and audio messages however, including advertisements that participants did not as strongly identify with or those that did not contain content relevant to participants’ society, culture, or interests, the large majority of participants reported that watching or listening to the advertisements would increase their likeliness of participating in safer sexual practices and would make them consider their risks. Possibly due to content- including those that participants did not strongly identifying with- invoking certain cognitive or emotional responses thus enabling mass media to target individual level behavior changes [33]. However, studies that have assessed media campaigns to promote HIV prevention have found mixed results regarding behavioral outcomes, particularly regarding increased condom use. We infer that the media content made participants in our study consider their sexual risks, however the impact may be short-lived. Several studies have reported small to moderate effects in increasing condom usage while others yielded insignificant effects; with some studies reporting increases in condom usage especially in countries with vigorous campaigns [26, 34]. Although the Project iMPPACS and PSA media campaigns did result in many of the women in our study considering the undertaking safer sexual practices, more research is needed to identify how to effectively bridge the thought or consideration of safer sex into actual practice or the uptake of safer sex behaviors. Further studies are needed to conduct more rigorous evaluation on the link or association between media effects and behavior changes [34].

### Future research

Future research should assess the specific components of media advertisements that can result in greater uptake of safe sex practices among this population. There is a need for future interventions focusing on the development of mass media campaigns for Black women in college, thus necessitating program developers to ascertain the features and content that Black women in college want to see included in HIV media messaging. Several participants in our study noted that many of the advertisements lacked content pertaining to their society, culture and interests. Additionally, a number of participants reported not strongly identifying with some of the advertisements. Despite this, participants were more likely to consider participating in safer-sex activities after listening to or viewing the advertisements. It is unclear as to whether the consideration of participating in safer-sex activities after watching or listening to advertisements results in the actual uptake of behaviors, and whether this effect can be sustained over the long term. It is also unclear as to whether there is an association between personally identifying with advertisement content and the uptake of safe sexual behavior practices. Thus, future research interventions should evaluate this. While we focused our study within an HBCU setting, there is a need for future research to evaluate similar interventions among Black female college students attending PWIs. Finally, many participants in our study reported watching or listening to social media more than radio advertisements. With the increased popularity and visibility of social media, there is a need for the development of mass media campaigns that target Black women in college through social media platforms.

### Limitations

Our study is not without limitations. First, non-probability sampling was used and our sample size was small consisting of students attending a southern HBCU. Thus, our results cannot be generalized to all Black female college students.

An essential element for future studies employing this method is to fully assess the relevance of demographic information related to socio-economic status, religiosity, social factors that may impact media preference and availability, previous exposure to HIV or treatment for an STI, and the emotional states of the participants before, during and after media exposure. In the context of a proof-of-concept, we a confident that this approach will yield useful information for effective messages.

In addition, given our sampling framework, there was potential for the presence of selection bias. Also, although perception analyzers were used to obtain an objective measure for participant responses and to limit response bias, self-reported behaviors and perceptions regarding HIV and sexual health is a highly sensitive topic. Thus, there was potential for reporting bias; perception analyzers were used to mitigate this. It can be inferred that higher perception values correspond to greater satisfaction and appeal of message content and that lower values correspond with decreased satisfaction of message content. However due to the study being a proof of concept, we cannot directly extrapolate impact. Furthermore, Project iMPPACS’s messages and PSAs were not specifically developed for Black female college students, and originally targeted Black teens in urban communities. However, this media content was chosen given that it was intended for Black youth and contained aspects that could be applicable to Black female college students [23]. Thus, we determined that this was an appropriate model to use for our study.

## Conclusions

In developing effective HIV prevention campaigns for Black women in college, it is still imperative for media campaigns to include content that is engaging and relatable through the incorporation of socially and culturally appealing elements. Media that includes linguistically tailored messages, recognizable backgrounds and contexts (e.g., a college or university environment), and content that represents the lived experiences of this priority population, are urgently needed. Effective media messaging can increase Black women’s perceived HIV risk and can result in the uptake of certain health promotion behaviors. Reducing HIV burdens among Black women in college will involve a multidisciplinary approach, and HIV prevention research focusing on tailored media messaging for this population should be considered as a public health strategy.

## Supplementary Information

**Additional file 1:** **Figure 1. **Perception analyzer real-time data analysis.

**Additional file 2:** **Table 1.** Example scene description, “dial-up”/”dial-down”playback intervals and scripted narration/dialogue (during interval) for a selection of Project iMPPACS Media Advertisements and audio. 

## Data Availability

The data that support the findings of this study are available within the article. Further inquiries regarding data can be made to the corresponding author.

## Abbreviations

HBCU: Historically Black Colleges and Universities
STI: Sexually Transmitted Infection
HIV: Human Immunodeficiency Virus
PSAs: Public Service Announcements
PWI: Predominantly White University
