# Supplementary figures and images for: Innovative perception analysis of HIV prevention messaging for black women in college: a proof of concept study
Source: BMC Public Health. 2022 Jun 25;22:1255. doi: 10.1186/s12889-022-13564-4 (PMC9233782; doi:10.1186/s12889-022-13564-4)

Additional file 1

Figure 1. Perception analyzer real-time data analysis


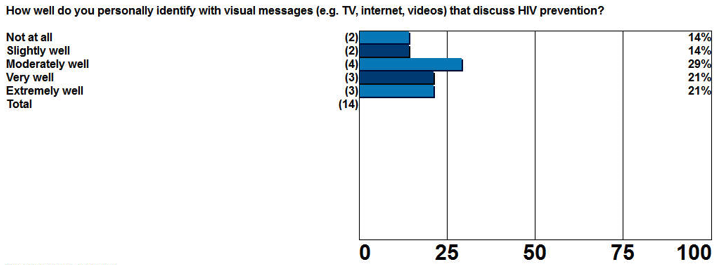

Supplement: Supplementary file 1 — Additional file 1: Figure 1. Perception analyzer real-time data analysis. [file 12889_2022_13564_MOESM1_ESM.docx]
