# Supplementary material for: Innovative perception analysis of HIV prevention messaging for black women in college: a proof of concept study
Source: BMC Public Health. 2022 Jun 25;22:1255. doi: 10.1186/s12889-022-13564-4 (PMC9233782; doi:10.1186/s12889-022-13564-4)

Additional file 2

Table 1: Example scene description, “dial-up”/”dial-down” playback intervals and scripted narration/dialogue (during interval) for a selection of PSA and Project iMPPACS Media Advertisements


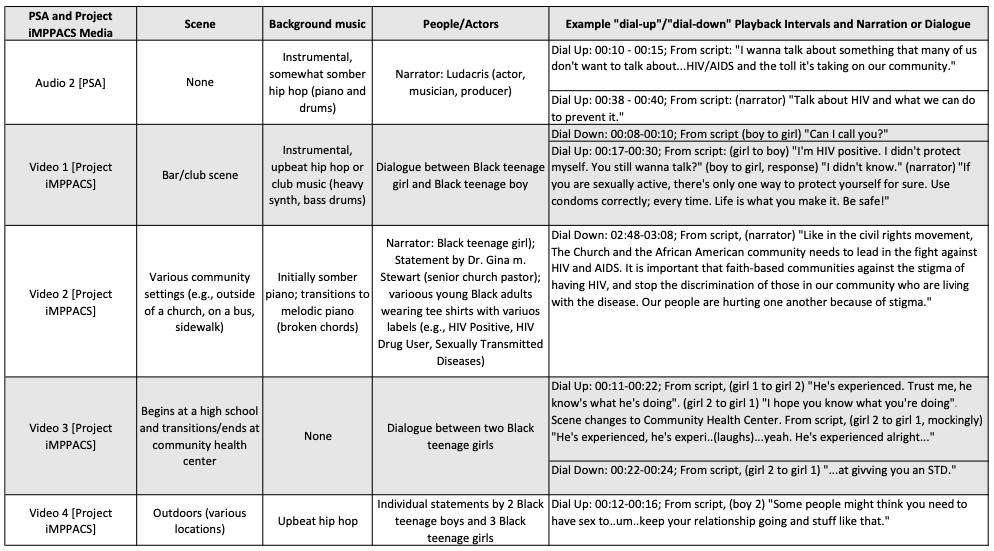

Supplement: Supplementary file 2 — Additional file 2: Table 1. Example scene description, “dial-up”/”dial-down”playback intervals and scripted narration/dialogue (during interval) for a selection of Project iMPPACS Media Advertisements and audio. [file 12889_2022_13564_MOESM2_ESM.docx]
